# Reduction in Blood Culture Contamination Through Use of Initial Specimen Diversion Device

**DOI:** 10.1093/cid/cix304

**Published:** 2017-05-17

**Authors:** Mark E. Rupp, R. Jennifer Cavalieri, Cole Marolf, Elizabeth Lyden

**Affiliations:** 1 Division of Infectious Diseases, and; 2 Department of Epidemiology, University of Nebraska Medical Center, Omaha

**Keywords:** blood culture, contamination, initial specimen diversion device.

## Abstract

**Background.:**

Blood culture contamination is a clinically significant problem that results in patient harm and excess cost.

**Methods.:**

In a prospective, controlled trial at an academic center Emergency Department, a device that diverts and sequesters the initial 1.5–2 mL portion of blood (which presumably carries contaminating skin cells and microbes) was tested against standard phlebotomy procedures in patients requiring blood cultures due to clinical suspicion of serious infection.

**Results.:**

In sum, 971 subjects granted informed consent and were enrolled resulting in 904 nonduplicative subjects with 1808 blood cultures. Blood culture contamination was significantly reduced through use of the initial specimen diversion device™ (ISDD) compared to standard procedure: (2/904 [0.22%] ISDD vs 16/904 [1.78%] standard practice, *P* = .001). Sensitivity was not compromised: true bacteremia was noted in 65/904 (7.2%) ISDD vs 69/904 (7.6%) standard procedure, *P* = .41. No needlestick injuries or potential bloodborne pathogen exposures were reported. The monthly rate of blood culture contamination for all nurse-drawn and phlebotomist-drawn blood cultures was modeled using Poisson regression to compare the 12-month intervention period to the 6 month before and after periods. Phlebotomists (used the ISDD) experienced a significant decrease in blood culture contamination while the nurses (did not use the ISDD) did not. In sum, 73% of phlebotomists completed a post-study anonymous survey and widespread user satisfaction was noted.

**Conclusions.:**

Use of the ISDD was associated with a significant decrease in blood culture contamination in patients undergoing blood cultures in an Emergency Department setting.

**Clinical Trials Registration.:**

NCT02102087.

## Summary

Blood culture contamination is a clinically significant problem that can be reduced through the use of an innovative device that diverts and sequesters the initial 1.5–2 mL of blood that contains the skin cells and microbes that result in contamination.

**(See the Editorial Commentary by McAdam on pages 206–7.)**

Blood cultures are frequently obtained in the care of patients with serious infections to detect bacteremia and fungemia and guide specific antimicrobial therapy. Unfortunately, contamination rates routinely range from 0.6% to 6%, resulting not infrequently in unnecessary antibiotic treatment and added laboratory expense [1]. False-positive blood cultures increase laboratory costs by approximately 20%, are associated with a nearly 40% increase in antibiotic charges, are treated with antimicrobials up to one half of the time, extend the length of hospital stay by up to 5 days, and subject patients to the real harms associated with antibiotic exposure such as toxicity, adverse effects, interactions, and emergence of resistance [2–7]. Because of their clinical significance, great efforts have been expended to limit false-positive blood cultures including the use of various skin disinfectants, trained phlebotomy teams, blood culture kits, needle exchange systems, culture bottle disinfection protocols, use of sterile gloves, and other programmatic attempts to limit contamination [1, 2, 8, 9]. Contamination of blood cultures is thought to be due in part to skin fragments colonized with bacteria that are dislodged with venipuncture [10]. The purpose of this project was to test a device that diverts and sequesters the first 1.5–2 mL portion of blood, which presumably carries the contaminating skin fragments, from the culture specimen to determine whether blood culture contamination is diminished [11].

## METHODS

### Study Design

Single center, prospective, controlled, open label trial. This study was reviewed and approved by the UNMC Institutional Review Board. This trial was registered at Clinicaltrials.gov (NCT 02102087).

### Setting

Emergency department and trauma center in an urban 689-bed university hospital.

### Test Device

Initial specimen diversion device (ISDD) (SteriPath®, Magnolia Medical Technologies), a pre-assembled, sterile blood culture system designed to divert and sequester the initial 1.5 to 2.0 mL of blood prior to culture bottle inoculation (ISDD diagram available in online supplemental materials).

### Subjects

Adult patients requiring phlebotomy for blood culture due to clinical suspicion of serious infection.

### Procedures

From November 2014 through October 2015, a convenience sample of subjects granting informed consent had paired blood cultures obtained by trained phlebotomists. Prior to venipuncture, the skin was disinfected with 2% chlorhexidine gluconate and 70% alcohol for 30 seconds and allowed to dry. The first blood sample (20 mL) was procured using standard procedures in which blood was drawn into a syringe and then injected into blood culture vials. The second blood sample (20 mL) was obtained using the ISDD in which the initial 1.5–2.0 mL of blood was diverted into a holding chamber and, upon activation, the rest of the sample was directed into the blood culture vials. In general, paired cultures were obtained from opposite arms. Standard aseptic practices were followed, and the blood culture vial tops were cleansed with 70% isopropyl alcohol pads before blood inoculation. Blood cultures were monitored using the BACTEC 9240 system (Becton Dickinson) with positive bottles being further characterized by film array (Biofire) and automated identification and susceptibility testing (Microscan). A culture was defined as contaminated if one or more of the following skin-residing organisms was recovered from only one of the paired cultures: coagulase-negative staphylococci, *Propionibacterium* sp., *Micrococcus* sp., viridians group streptococci, *Corynebacterium* sp., or *Bacillus* sp.

### Survey

An anonymous 7-question post-study survey was distributed to the phlebotomists who used the device to gather qualitative information about user experience (survey form available in online supplemental materials).

### Statistical Analysis

McNemar’s test was used to compare proportions of blood cultures that were contaminated as well as those yielding true pathogens. Fisher exact test was used to examine the association between individual phlebotomists and contamination rate. The time-to-detection was compared using the paired *t*-test. In a post hoc analysis of blood cultures obtained in the emergency department, the rate of blood culture contamination for all cultures (phlebotomist-drawn and nurse-drawn cultures for all patients—those in the study and those not enrolled), as defined by a standard laboratory definition maintained by the hospital clinical microbiology laboratory, was compared for 3 time periods: 6 months prior to initiation of the project, 12-month intervention period, and 6-month post intervention. Poisson regression was used to model the rate of contamination per month as a function of intervention.

## RESULTS

**Figure 1.**
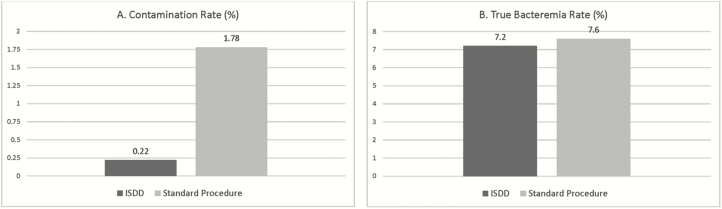
Performance of ISDD vs standard procedure. *A*, Contamination rate. *B*, Detection of true bacteremia. Abbreviation: ISDD, initial specimen diversion device.

Nine-hundred seventy-one subjects granted informed consent and were enrolled resulting in 904 subjects with 1808 blood cultures after exclusion of repeat patients. Fifty-five percent of the subjects were male, and the mean age was 59 years. In sum, 152/1808 (8.4%) of the blood cultures yielded microbial growth with 134/1808 (7.4%) regarded as true pathogens and 18/1808 (1%) regarded as contaminants. The ISDD was associated with less blood culture contamination compared to standard procedure (Figure 1): (2/904 [0.22%] vs 16/904 [1.78%], *P* = .001). Sensitivity was not compromised: true septicemia was noted in 65/904 (7.2%) with ISDD and 69/904 (7.6%) with standard procedure, *P* = .41 (Figure 1). The time to detection did not vary between the 2 methods. The mean time to detection for the standard procedure method was 13.68 hours (SD 8.43 hours) compared to 15.60 hours (SD 13.93 hours) for the ISDD (*P* = .16). The ratio of true positive to false positive culture for ISDD was 33:1 (65/2) and for standard procedure 4.3:1 (69/16); the likelihood of a positive culture being a true positive for ISDD was 97% (65/67) and for standard procedure, 81% (69/85). No association was noted between the 11 individual phlebotomists and contamination using ISDD (*P* = .62) or standard procedure (*P* = .31). If all subjects are included in the analysis (N = 971 subjects; 1942 blood cultures) the results do not differ with the contamination rate being 0.21% (ISDD) vs 1.65% (standard procedure), and the true septicemia rate being 7.2% (ISDD) vs 7.3% (standard procedure). No needlestick injuries or potential bloodborne pathogen exposures were reported.

Microbiologic results are fully characterized in (Table 1). The most common cause of true bacteremia were the *Enterobacteriaceae* (39%) followed by *S. aureus* (20.7%). Blood culture contamination was noted in 16 of the standard procedure cultures and was most prominently caused by coagulase-negative staphylococci (50%) and *Propionibacterium* sp. (19%), whereas only 2 of the ISDD cultures were contaminated. Discordance between the 2 methods of phlebotomy with regard to detection of true bacteremia was essentially equal (Table 1). In 12 instances, the standard procedure yielded a true pathogen while the ISDD was sterile, and in 11 instances, the ISDD yielded a true pathogen while the standard procedure was sterile. The bacterial species involved in the discordant true bacteremia results were similar between the 2 phlebotomy methods (Table 1). The 8 cases in which common skin flora were recovered from both the ISDD and standard procedure cultures were further reviewed. Four cases each were due to coagulase- negative staphylococci and viridans streptococci, respectively. In 7 of the cases, Infectious Diseases consultation was obtained at the time of bacteremia, and in all 7, the bloodstream isolate was regarded as the result of an infection. Bacteremia due to normal skin flora was secondary to the following conditions: endocarditis (2), left ventricular assist device infection (1), orthopedic hardware infection (1), cholangitis (1), urosepsis (1), infected decubitus ulcer (1). In the case not seen by an Infectious Diseases expert, the patient experienced a colonic perforation and had viridans streptococci recovered from blood and peritoneal fluid. For the purposes of the study analysis, all of these subjects were regarded as having true bacteremia.

**Table 1.** Microbiology Results

	ISDD	Standard Practice
Contamination	N = 2 Coagulase-negative staphylococci (1), *Corynebacterium* sp. (1)	N = 16 Coagulase negative-staphylococci (8), *Propionibacterium* sp. (3), viridans streptococci (2), *Micrococcus* sp. (2), *Corynebacterium* sp. (1)
True bacteremia	ISDD & Standard Practice^a^ (N = 143)	
	*Enterobacteriaceae* (42.7%) [*E. coli* (29.4%), *Klebsiella* sp. (6.3%), *Proteus* sp. (2.8%), *Morganella* sp. (1.4%), *Salmonella* sp. (1.4%), *Enterobacter* sp. (0.7%), *Serratia* sp. (0.7%)], *S. aureus* (21%), *S. pneumoniae* (7.7%), *Enterococcus* sp. (5.6%), viridans streptococci (5.6%), coagulase-negative staphylococci (5.6%), *S. pyogenes* (3.5%), *H. influenzae* (2.1%), <2% each caused by *Pseudomonas aeruginosa*, *S. agalactiae*, *Veillonella* sp., *Candida* sp., and *Peptostreptococcus* sp.	
Discordant cultures	ISDD (+)/Standard practice (−) (N = 11)	ISDD (−)/Standard practice (+) (N = 12)
	*Klebsiella* sp. (4)*, S. aureus* (2), *Enterococcus* sp. (2), *S. pneumoniae* (1), *E. coli* (1), *Peptostreptococcus* sp.(1)	*E. coli* (2), *S. aureus* (2), *S. pneumoniae* (2), *Morganella* sp. (2), *S. pyogenes* (1), *Enterobacter* sp. (1), *H. influenzae* (1), *C. albicans* (1)

Abbreviation: ISDD, initial specimen diversion device.

^a^4 subjects had polymicrobic blood cultures.

In the post hoc analysis, the rate of blood culture contamination for cultures obtained by phlebotomists and nurses in the emergency department during the intervention period was compared to the contamination rate in the 6-month periods preceding and following. The rate of contamination was significantly decreased during the intervention amongst the phlebotomists but was not significantly different for nurses (who did not use the ISDD) (Table 2).

**Table 2.** Poisson Regression Analysis of Blood Culture Contamination Rate in the Emergency Department for the Intervention Period (Nov 2014–Oct 2015) Compared to the Preceding and Following 6-Month Periods for All Patients Who Underwent Blood Culture

Observation Period	Contamination Rate /1000 Blood Cultures		
	Observed Rate	Model Estimated Risk vs Intervention (95% CI)	P value
All blood cultures			
Pre 6 mo (N 3454)	33.01	1.356 (1.068–1.723)	.013
Intervention (N 6697)	24.34	Reference	
Post 6 mo (N 3596)	28.09	1.154 (.90–1.48)	.258
Phlebotomist-drawn blood cultures			
Pre 6 mo (N 2684)	26.45	1.305 (.967–1.76)	.082
Intervention (N 5326^a^)	20.28	Reference	
Post 6 mo (N 2905)	28.23	1.392 (1.045–1.855)	.024
Nurse-drawn blood cultures			
Pre 6 mo (N 770)	55.84	1.39 (.933–2.071)	.106
Intervention (N1369)	40.18	Reference	
Post 6 mo (N 435)	32.04	0.797 (.444–1.434)	.45

Abbreviation: CI, confidence interval.

^a^Blood cultures from all patients (those enrolled in the study as well as those who underwent blood culture by standard procedures and were not enrolled in the study) were analyzed.

Eight of 11 (73%) of phlebotomists returned a completed survey. Phlebotomists related that the ISDD was easy to use, and a widely perceived advantage was the ability to easily draw additional tubes of blood in addition to the sample for culture and the lack of need to transfer blood from a syringe to blood culture vials. Most phlebotomists noted that they purposely avoided use of the ISDD for uncooperative patients or those who were perceived to be a “hard stick” due to small or fragile veins.

## DISCUSSION

We observed a significant decrease in blood culture contamination associated with use of the ISDD. Contamination is thought to be due to bacteria that are carried on skin cells that are dislodged with venipuncture [10] and the ISDD functions by diverting and sequestering the first 1.5–2 mL of blood, which contain the contaminants, away from the rest of the sample to be cultured [11]. Although initial specimen diversion is not widely used to prevent contamination of blood cultures, it is a standard technique in blood banking and has been successfully employed worldwide to minimize the risk of microbial contamination of blood components [12]. Reassuringly, no decrease in detection of true bacteremia was noted in our study. The discordant detection of true bacteremia between the 2 methods of phlebotomy was essentially equal (12 subjects with (+) standard procedure blood culture and (−) ISDD culture vs 11 subjects with (+) ISDD blood culture and (−) standard procedure culture), which emphasizes the need for 2 sets of blood cultures in order to adequately detect true bacteremia. Lee et al noted that only 73% of episodes of true bacteremia were detected with a single blood culture [13]. Phlebotomists noted that the ISDD was easy to use, and it was versatile in collecting blood for both cultures and other laboratory tests. There were no reported needle stick injuries or potential blood borne pathogen exposures.

A large number of techniques can be used to limit blood culture contamination, and the ISDD should be used to complement other approaches. First, personnel performing blood cultures should be well trained and dedicated to the task [4, 14]. The skin should be disinfected prior to venipuncture, but the optimum choice of disinfectant continues to be debated [15, 16]. Drawing blood for cultures from indwelling catheters should be avoided unless the catheter is thought to be the source of bacteremia [17]. Strict aseptic practices must be maintained throughout the process of phlebotomy and transfer of blood to culture vials [18, 19] and use of a prepackaged blood culture kit has proven useful in reducing contamination in a variety of settings [2, 20]. Finally, monitoring the rate of contamination and reporting surveillance data regularly to phlebotomists is recommended [2]. Unfortunately, none of these preventative measures will eliminate blood culture contamination due to the microbes that survive local skin disinfection and are inadvertently included in the blood specimen with dislodged dermal cells. In our emergency department, where most of the contamination prevention measures described above are routinely employed, the baseline rate of blood culture contamination in the standard lab procedure cultures was 1.78%, well within the target of <3% that is generally regarded as the expected rate of contamination when blood cultures are properly performed [21, 22]. This already low rate of contamination was further reduced to 0.22% with the use of the ISDD. Syringe withdraw of venous blood (usually 20 mL) for culture has long been a routine practice. However, the mixing of blood in the syringe barrel is likely to distribute skin fragments in the 2 portions (10 mL each) for the aerobic and anaerobic mediums resulting in recovery of microbes in both bottles and erroneously suggesting a high concentration of skin residing organisms.

The post hoc analysis of the rate of blood culture contamination in the emergency department further documents the impact of the ISDD and assesses for secular trends in the contamination rate. A higher rate of contamination was observed for the phlebotomists (who used the ISDD during the intervention period) in both the pre- and post-periods compared to the intervention period. The rate of contamination for nurse-drawn blood cultures (who did not use the ISDD) did not vary significantly over the 3 evaluation periods. Interestingly, although the rate of contamination of nurse-drawn cultures remained consistently higher than that associated with phlebotomist-drawn cultures, the contamination rate for nurses did appear to decrease over the course of the intervention period and post-intervention period. We hypothesize this decrease was due to spill-over (ie, our work with the phlebotomists in the emergency department increased the awareness of proper technique and the clinical impact of blood culture contamination with the emergency department nurses). We are not aware of any programmatic changes in nurse blood culture practices.

Blood culture contamination is a clinically significant problem that results in extra expense and patient harm. Alahmadi et al noted in a case-control study in the United Kingdom that blood culture contamination was associated with 5.4 extra hospital days at a cost of approximately $7500 USD [7]. Similarly, in the United States, Gander and colleagues observed excess charges of $8720 per contamination event [4]. Blood culture contamination is associated with unneeded antibiotic treatment in approximately 30%–40% of patients [3, 5, 6]. Although rapid blood culture technology, such as nucleic acid amplification assays, enables laboratories to greatly reduce the time needed to identify various microbes, thus limiting misinterpretation and unneeded tests and antimicrobics, these rapid techniques are often very costly. Therefore, costs associated with efforts to reduce blood culture contamination can be highly cost effective, even in the era of rapid diagnostics. Although a rigorous cost-effectiveness analysis is beyond the scope of this project and is speculative, we noted in the 6 months before and after our project a contamination rate of 2.6% (N = 407) stemming from the 15 442 blood cultures obtained by phlebotomist throughout the hospital. If the low rate of contamination that we observed in the study (0.22%) was applied to all blood cultures obtained by phlebotomists, it would equate to 373 prevented episodes of contamination. The costs associated with blood culture contamination range from $1000 (1998) to $8700 (2009) per case [4, 5]. If a midpoint cost estimate is used ($4850), and the added cost of the device is not taken into account, it equates to a cost avoidance of 1.8 million dollars per year at our institution.

Our study has several important limitations that should be noted. First, the study was conducted at a single center in an emergency department, and thus questions regarding generalizability are valid. Similarly, a convenience sample of patients was studied (approximately 36% of the total number of blood cultures obtained by phlebotomists in the emergency department during the intervention period were included in the primary analysis). The convenience sampling may have led to selection bias. Most of the phlebotomists noted that they avoided use of the ISDD for uncooperative patients or those with poor vascular access. Consecutive patient inclusion was made more difficult by the IRB-imposed requirement for informed consent—thus excluding incompetent patients and those requiring immediate attention (ie, limited time to obtain informed consent). It could be argued that in a nonsignificant risk study, informed consent should not be required to employ an FDA-registered and listed device [http://www.accessdata.fda.gov/scripts/cdrh/cfdocs/cfRL/rl.cfm?lid=347659&lpcd=KST] in the manner in which the device is intended to be used. Legitimate questions remain with regard to broader use of the ISDD and whether it would function in the same manner when used for all patients. Also, the ISDD was compared to our standard practice that involved phlebotomy via a syringe and subsequent transfer of blood to culture vials rather than a direct inoculation to medium system. Future studies could involve comparison of ISDD vs. direct to medium blood culture systems. Finally, the 1.5 to 2.0 mL of blood sequestered by the ISDD might prove to be problematic in patients with very low blood volume, such as neonates. However, we have no reason to believe ISDD would not reduce blood culture contamination with broader application in most other patient populations.

In conclusion, use of the ISDD resulted in a significant reduction in blood culture contamination to a very low rate of 0.22% in a convenience sample of emergency department patients. Reduced blood culture contamination should result in decreased costs and improved clinical outcome measures. The ISDD should be further studied in a broader sampling of consecutive patients including children. The very low rate of contamination observed in our study may justify abandonment of the current practice of performing 2 separate venipunctures (in order to better rule out contamination), which would result in improved patient satisfaction and healthcare provider safety (fewer venipunctures). In addition, use of the ISDD to draw blood from vascular catheters should be explored to see if similar blood culture contamination reduction is observed.

## Supplementary data

Supplementary materials are available at *Clinical Infectious Diseases* online. Consisting of data provided by the authors to benefit the reader, the posted materials are not copyedited and are the sole responsibility of the authors, so questions or comments should be addressed to the corresponding author.

## Supplementary Material

Phlebotomist_Survey

ISDD_SteriPath_w_21G_NS

## Notes

***Acknowledgments.*** The authors express their appreciation to the Emergency Department phlebotomy team for their dedicated service and to Ms Sandy Nelson for secretarial support.

***Financial support.*** This work was supported by Magnolia Medical Technologies, Inc., in the form of a research contract to the University of Nebraska Medical Center.

***Potential conflicts of interest.*** M. E. R. has received research funding and has served as a consultant for 3M. All other authors have no conflicts. All authors have submitted the ICMJE Form for Disclosure of Potential Conflicts of Interest. Conflicts that the editors consider relevant to the content of the manuscript have been disclosed.
