# Supplementary material for: Reduction in Blood Culture Contamination Through Use of Initial Specimen Diversion Device
Source: Clin Infect Dis. 2017 May 17;65(2):201–5. doi: 10.1093/cid/cix304 (PMC5849098; doi:10.1093/cid/cix304)
Supplement: Phlebotomist_Survey [file cix304_suppl_phlebotomist_survey.docx]

Quality Assurance Survey

Please give us your feedback on the diversion device.

1. What did you like about the diversion device?
2. What did you not like about the diversion device?
3. How long did it take you to feel comfortable with using the device?
4. After you were familiarized with the device, did it take a longer period of time to perform blood culture collections with the device?
5. Was the device difficult to manipulate?
6. Were there any patients for whom you avoided using the device?
7. Did patients make any comments to you about the device?

Please return this survey in the envelope provided. Thanks very much!
