# Supplementary figures and images for: Reduction in Blood Culture Contamination Through Use of Initial Specimen Diversion Device
Source: Clin Infect Dis. 2017 May 17;65(2):201–5. doi: 10.1093/cid/cix304 (PMC5849098; doi:10.1093/cid/cix304)

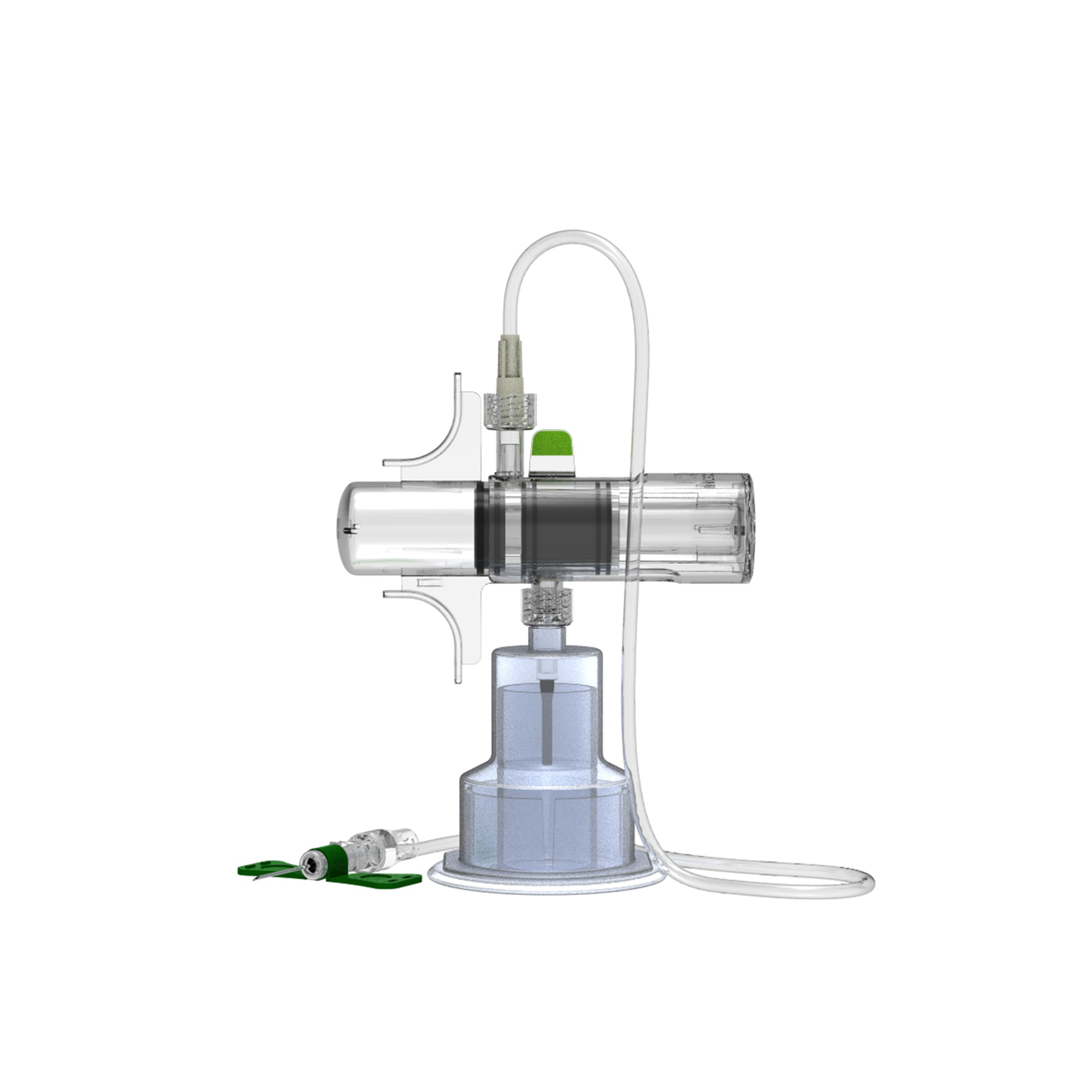

Supplement: ISDD_SteriPath_w_21G_NS [file cix304_suppl_isdd_steripath_w_21g_ns.png]
